# A novel small open reading frame gene, *IbEGF*, enhances drought tolerance in transgenic sweet potato

**DOI:** 10.3389/fpls.2022.965069

**Published:** 2022-10-31

**Authors:** Yuanyuan Zhou, Hong Zhai, Shihan Xing, Zihao Wei, Shaozhen He, Huan Zhang, Shaopei Gao, Ning Zhao, Qingchang Liu

**Affiliations:** Key Laboratory of Sweetpotato Biology and Biotechnology, Ministry of Agriculture and Rural Affairs/ Beijing Key Laboratory of Crop Genetic Improvement/Laboratory of Crop Heterosis and Utilization, Ministry of Education, College of Agronomy & Biotechnology, China Agricultural University, Beijing, China

**Keywords:** sweet potato, sORF, *IbEGF*, drought tolerance, phytohormone

## Abstract

Small open reading frames (sORFs) can encode functional polypeptides or act as *cis*-translational regulators in stress responses in eukaryotes. Their number and potential importance have only recently become clear in plants. In this study, we identified a novel sORF gene in sweet potato, *IbEGF*, which encoded the 83-amino acid polypeptide containing an EGF_CA domain. The expression of *IbEGF* was induced by PEG6000, H_2_O_2_, abscisic acid (ABA), methyl-jasmonate (MeJA) and brassinosteroid (BR). The IbEGF protein was localized to the nucleus and cell membrane. Under drought stress, overexpression of *IbEGF* enhanced drought tolerance, promoted the accumulation of ABA, MeJA, BR and proline and upregulated the genes encoding superoxide dismutase (SOD), catalase (CAT) and peroxidase (POD) in transgenic sweet potato. The IbEGF protein was found to interact with IbCOP9-5α, a regulator in the phytohormone signalling pathways. These results suggest that IbEGF interacting with IbCOP9-5α enhances drought tolerance by regulating phytohormone signalling pathways, increasing proline accumulation and further activating reactive oxygen species (ROS) scavenging system in transgenic sweet potato.

## Introduction

Drought stress is the most complex and devastating factor of abiotic stresses and seriously affects the productivity of agricultural crops in the world (Shinozaki and Yamaguchi-Shinozaki, 2007; Ceccarelli et al., 2010; Zhu, 2016; Peterson et al., 2021). Improving the drought tolerance of agricultural crops has become important for the sake of world food security (Zhu, 2016). The plant hormones, such as abscisic acid (ABA), jasmonic acid (JA), brassinosteroid (BR), ethylene (ETH) and salicylic acid (SA) play important roles in the response of plants to drought stress (Fujita et al., 2005; Vargas et al., 2014; Jin et al., 2016; Xu et al., 2017). The biochemical and genetic studies have revealed that peptides are new signalling molecules in plants (Pearce et al., 2001; Ryan and Pearce, 2001; Lindsey et al., 2002; Ryan et al., 2002). Until the initial discovery of the 18-amino acid (aa) polypeptide defense hormone system in tomato leaves in 1991, polypeptides are thought to be a new class of plant hormones (Pearce et al., 1991).

Small open reading frames (sORFs), which encode polypeptides of less than 100 aa in eukaryotes and 50 aa in prokaryotes, have been historically excluded from genome annotation (Basrai et al., 1997; Frith et al., 2006; Hanada et al., 2013). However, recent studies have revealed thousands of translated sORFs in genomes spanning evolutionary space (Khitun et al., 2019). These sORFs can code for functional polypeptides or act as *cis*-translational regulators, including stress proteins, energy metabolism-related proteins, mating pheromones, hormones, transcriptional regulators, nucleases, transporters etc. (Basrai et al., 1997; Kastenmayer et al., 2006; Khitun et al., 2019; Kim et al., 2021).

The first systematic investigation of sORFs function was conducted in yeast. It was showed that 299 sORFs existed in the yeast genome, representing approximately 5% of the annotated ORFs, and 22 of them was required for haploid growth, growth in the presence of DNA damage and replication-arrest agents, growth at high temperature or growth in the presence of a nonfermentable carbon source (Kastenmayer et al., 2006). More than 7000 sORFs have been identified in *Arabidopsis*, and 3241 of them likely belong to novel coding genes in the *Arabidopsis* genome (Hanada et al., 2007).

A number of specific polypeptides encoded by sORFs have been identified in plants. In tomato, rapid alkalinization factor (RALF), which is a 5-kDa ubiquitous polypeptide, arrested root growth and development (Pearce et al., 2001). The 23-aa peptide AtPep1 increased root development and enhanced resistance to *Pythium irregulare* in transgenic *Arabidopsis* (Huffaker et al., 2006; Poncini et al., 2017). In rice, the plants overexpressing *OsDT11* encoding an 88-aa peptide displayed reduced water loss and stomatal density and enhanced drought tolerance (Li et al., 2017). The CLAVATA3/EMBRYO-SURROUNDING REGION-RELATED 25 (CLE25) peptide modulated stomatal control in *Arabidopsis* (Takahashi et al., 2018). The small peptide OsDSSR1 (*Oryza sativa* L. drought and salt stress response-1) enhanced drought tolerance in transgenic rice (Cui et al., 2018). A C-terminal peptide fragment (AtPep3) increased salt tolerance in transgenic *Arabidopsis* (Nakaminami et al., 2018). The CLAVATA3/EMBRYO SURROUNDING REGION (CLE) peptides are important ligands that have roles in regulating cell proliferation and differentiation in plant shoots, roots and other tissues (Li et al., 2019; Song et al., 2021). In *Arabidopsis*, CLE9 induced stomatal closure and enhanced drought tolerance (Zhang et al., 2019). PttCLE47 from *Populus* promoted cambial development and secondary xylem formation in hybrid aspen (Kucukoglu et al., 2020). The phytosulfokine (PSK) peptide regulated drought-induced flower drop in tomato (Reichardt et al., 2020).

Epidermal growth factor (EGF) is a short peptide with an important role in the migration and proliferation of cells (Cohen, 1983; Plata-Salaman, 1991; Ren et al., 2018). The calcium-binding EGF-like domain (EGF_CA domain) is a sequence of about 40 aa and it is present in numerous extracellular proteins and membrane-bound proteins (Rao et al., 1995). Some cell surface receptors have numerous EGF modules in tandem, and a subset of EGF modules bind one calcium ion (Ca^2+^) for the biosynthesis of biologically active proteins (Stenflo et al., 2000). The cysteine-rich epidermal patterning factor (EPF) and EPF-like (EPFL) regulate stomatal patterning in *Arabidopsis* (Hara et al., 2007; Jewaria et al., 2013; Zeng et al., 2020). The epidermal growth factor receptors (EGFR/ErbB) are membrane-anchored tyrosine kinases with a critical role in cell growth and development (Pascarelli et al., 2021). However, the EGF protein family with EGF_CA domain have not been functionally characterized in plants.

Sweetpotato, *Ipomoea batatas* (L.) Lam., is an important food crop worldwide and its productivity is seriously affected by drought stress (Zhu et al., 2022). Up to now, sORFs have not been identified in sweet potato. In this study, a novel gene coding for the 83-aa polypeptide, *IbEGF*, was cloned from sweet potato. The EGF protein contained an EGF_CA domain. Functional analysis showed that *IbEGF* enhanced drought tolerance in transgenic sweet potato. The underlying mechanism of this gene in drought tolerance of sweet potato was also analyzed.

## Materials and methods

### Plant materials

Sweetpotato line Xushu55-2 with drought tolerance was used for isolation and expression analysis of the *IbEGF* gene. Sweetpotato cv. Lizixiang was employed to characterize the function of *IbEGF*. The subcellular localization, bimolecular fluorescence complementation (BiFC) assay and co-immunoprecipitation (co-IP) assay of IbEGF were conducted using *Nicotiana benthamiana*.

### Cloning ands analysis of *IbEGF* and its promoter

Total RNA from the *in vitro*-grown Xushu55-2 plants was extracted using the Trozol Up Kit (ET111, Transgen, Beijing, China). The first-strand cDNA was transcribed from the total RNA with the PrimeScript™ RT reagent Kit with gDNA Eraser (PR047A, Takara, Beijing, China). The open-reading frame (ORF) of *IbEGF* was amplified from the first-strand DNA using the homologous cloning method and the expressed sequence tag (EST) database of Xushu55-2 (Zhu et al., 2018). Amino acid sequence alignment was analyzed using DNAMAN V6 software. The phylogenetic tree was constructed with MEGA 7.0 software with 1000 bootstrap replicates. The molecular weight and theoretical isoelectric point (*p*I) of IbEGF were calculated with ProtParam tool (https://web.expasy.org/protparam/).

Genomic DNA of the *in vitro*-grown Xushu55-2 plants was extracted using the cetyltrimethylammonium bromide (CTAB) method (Rogers and Bendich, 1985) for obtaining the genomic sequence and promoter sequence of *IbEGF* with the homologous cloning method (**Supplementary Table S1**). The exon-intron and *cis*-acting regulatory elements in its promoter were analyzed using the Spign tool (http://www.ncbi.nlm.nih.gov/sutils/spign/splign.cgi) and PlantCARE (http://bioinformatics.psb.ugent.be/webtools/plantcare/html/), respectively.

### Expression analysis

The transcript levels of *IbEGF* in leaf, stem and root tissues of the 4-week-old *in vitro*-grown plants and leaf, stem, hair root, pencil root and storage root tissues of the 80-day-old field-grown plants of Xushu55-2 were analyzed with quantitative real-time PCR (qRT-PCR) (Zhou et al., 2020). Furthermore, the 4-week-old *in vitro*-grown Xushu55-2 plants were stressed in Hoagland solution with H_2_O (control), 30% PEG6000, 100 μM H_2_O_2_, 100 mM ABA, 100 μM MeJA and 100 nM BR, respectively, and sampled at 0, 1, 3, 6, 12 and 24 h after stresses for analyzing the expression of *IbEGF. Ibactin* (AY905538) was used to normalize the expression levels in sweet potato (Liu et al., 2014). All the specific primers are showed in **Supplementary Table S1**.

### Subcellular localization

Using primers IbEGF-OS-F/R (**Supplementary Table S1**), the ORF of *IbEGF* without stop codon was amplified, and then inserted into pSuper1300 vector to produce green fluorescent protein (GFP) fusion construct pSuper1300-*IbEGF*. The fusion construct was transformed into the *Agrobacterium tumefaciens* strain EHA105, and then infiltrated *N. benthamiana* leaves. The empty pSuper1300 vector was used as a control. After 48 h of infiltration, the fluorescence signal was observed under excitation wavelength 488 nm using a laser scanning confocal microscope (Olympus, Tokyo, Japan). Meanwhile, the pSuper1300-*IbEGF* and pSuper1300 vectors were transferred into maize protoplasts, respectively. The empty pSuper1300 vector was used as a control. After 16 h of growth, the fluorescence signal was observed under a laser scanning confocal microscope.

### Transactivation activity assay

The full-length of *IbEGF* was constructed to the yeast expression vector pGBKT7. pGBKT7-*IbEGF*, pGBKT7-53 (positive control) and pGBKT7 (negative control) were separately transferred into the yeast strain Y2H Gold by the PEG/LiAc method. The transformed yeast strains were cultured on synthetic defined (SD) plates without tryptophan (SD/-trp) for 3 days and then cultured on SD plates containing 5-bromo-4-chioro-3-indoxyl-α-galactopyranoside (X-α-gal) but lacking tryptophan and histidine (SD/-Trp/-His/X-α-gal) for 3 days.

### Regeneration of the transgenic sweet potato plants

The overexpression vector pSuper1300-*IbEGF* was introduced into the *A. tumefaciens* stain EHA105. Embryogenic suspension cultures of sweet potato cv. Lizixiang were prepared as described by Liu et al. (2001). The transformation and plant regeneration were performed as previously described (Liu et al., 2014). The identification of the transgenic plants was conducted by PCR with specific primers (**Supplementary Table S1**). The expression levels of *IbEGF* in the *in vitro*-grown transgenic and wild type (WT) plants were analyzed using specific primers designed in the non-conserved domain (**Supplementary Table S1**). The transgenic plants were transferred to pots with soil, vermiculite and humus (1:1:1, v/v/v) in a greenhouse and further to a field for evaluating their drought tolerance.

### Drought tolerance assay

The *in vitro*-grown transgenic and WT plants were cultured on Murashige and Skoog (MS) medium with or without (control) 20% PEG6000 at 27 ± 1°C under 13 h of daylight at 54 μM m^–2^ s^–1^. After 4 weeks, the root length and fresh weight were measured (Ren et al., 2020). Furthermore, the 25-cm-long cuttings from the field-grown transgenic and WT plants were treated in Hoagland solution for 4 weeks (control) or in Hoagland solution with 20% PEG6000 for 2 weeks followed by 2 weeks of Hoagland solution in a greenhouse (Ren et al., 2020). The root number and fresh weight were measured.

For further evaluation of drought tolerance, the 25-cm-long cuttings from the field-grown transgenic and WT plants were planted in transplanting boxes with soil, vermiculite and humus (1:1:1, v/v/v) in a greenhouse. After 2 weeks of treatment with Hoagland solution, they were subjected to continuous drought stress for 8 weeks and the plants irrigated with Hoagland solution for 8 weeks were used as a control.

### Stomatal aperture assay

The leaves from greenhouse-grown transgenic and WT plants were treated in stomatal opening solution containing 50 mM KCl, 10 mM MES-KOH and 10 mM CaCl2 (pH 6.1) for 3 h and then incubated in stomatal opening solution with 20 μM ABA for 2 h. Randomly selected 80 stomata were measured using a fluorescence microscope (Revolve, Echo, USA).

### Measurement of leaf water loss rate

The leaves from greenhouse-grown transgenic and WT plants were placed at the room temperature. These leaves were weighed hourly (0 h-8 h) for calculating the rate of leaf water loss.

### Analyses of components and gene expression in response to drought stress

The leaves of the transgenic and WT plants subjected to drought stress for 4 weeks in transplanting boxes were used to analyze the components and gene expression in response to drought stress. The ABA, MeJA and BR contents were analyzed with indirect enzyme linked immune sorbent assay (ELISA) kit (Suzhou Comin Biotechnology Co., Ltd., China). The proline, malondialdehyde (MDA) and H_2_O_2_ contents and superoxide dismutase (SOD) activity were measured with Assay Kit (Suzhou Comin Biotechnology Co., Ltd., China). The expression levels of the constitutive photomorphogenesis 9-5*α* (*COP9-*5*α*) gene, a regulator in the phytohormone signalling pathways, and genes encoding SOD, catalase (CAT), and peroxidase (POD) of reactive oxygen species (ROS) scavenging system were analyzed with qRT-PCR (**Supplementary Table S1**) according to the method of Liu et al. (2014).

### Yeast two-hybrid assay

After the transactivation activity assay, the *IbEGF* without transactivation activity domain was used as the bait to transform Y2H Gold for screening for the sweet potato yeast two-hybrid library following Yeast Protocols Handbook (Clontech). The co-transformed yeast strain was plated onto SD/-Ade/-His/-Leu/-Trp medium containing 3 mM 3-aminotriazole (3-AT) to screen for potential interaction proteins. pGBKT7-53 and pGADT7-T were co-transformed into Y2H Gold as positive control.

### BiFC Assay

The coding sequence (CDS) of *IbEGF* was ligated into pSPYNE-35S vector with the N-terminus of yellow fluorescent protein (nYFP) and the interacted protein gene was constructed into pSPYCE-35S vector with the C-terminus of YFP (cYFP). The two vectors were separately transformed into *A. Tumefaciens* strain GV3101, and subsequently coinjected into *N. benthamiana* leaves. After 2 days, the YFP signals were observed (Zhou et al., 2020).

### Co-IP assay

The Myc-IbEGF and IbCOP9-5α-GFP vectors were transiently expressed in *N. benthamiana* leaves. Total proteins were extracted from the leaves with extraction buffer, mixed with Myc magnetic beads (P2118, Beyotime) and then incubated at 4°C for 4 h (Zhang et al., 2020). The agarose was washed at least five times with extraction buffer and boiled in 5× SDS loading buffer for 15 min to separate the proteins from the agarose beads. The proteins were detected using polyclonal anti-Myc (1:10000, BE2011, EASYBIO) and anti-GFP antibodies (1:10000, BE2002, EASYBIO).

### Statistical analysis

All experiments were done for three biological replicates. Data were presented as the mean ± SE and analyzed using Student’s *t*-test (two-tailed analysis). Significance levels at *P* < 0.05 and *P* < 0.01 were denoted by ∗ and ∗∗, respectively.

## Results

### Cloning and sequence analysis of *IbEGF* and its promoter

The novel *IbEGF* gene was isolated from sweet potato line Xushu55-2. The 252-bp CDS sequence of *IbEGF* encoded a polypeptide of 83 aa with a molecular weight of 8.68 kDa and a predicted *p*I of 10.54. *IbEGF* consisted of one single exon. The EGF protein contained an EGF_CA domain (**Supplementary Figure S1A**). This protein showed high sequence identity with EGF proteins from *Trema orientale* (PON94549.1, 65.06%), *Actinidia chiensis* var. *chinensis* (PSS20878.1, 63.10%), *Morus natabilis* (EXB37025.1, 61.90%), *Manihot esculenta* (OAY53736.1, 60.92%), *Theobroma cacao* (EOY30817.1, 60.00%) and *Nicotiana attenuate* (OIT39415.1, 57.47%) (**Supplementary Figure S1B**). The 1591-bp promoter region of *IbEGF* contained several phytohormone or stress-responsive *cis*-acting regulatory elements, including AuxRE, ABRE, ARE, and LTR (**Supplementary Figure S2**).

### Expression analysis of *IbEGF* in sweet potato

**Figure 1:**
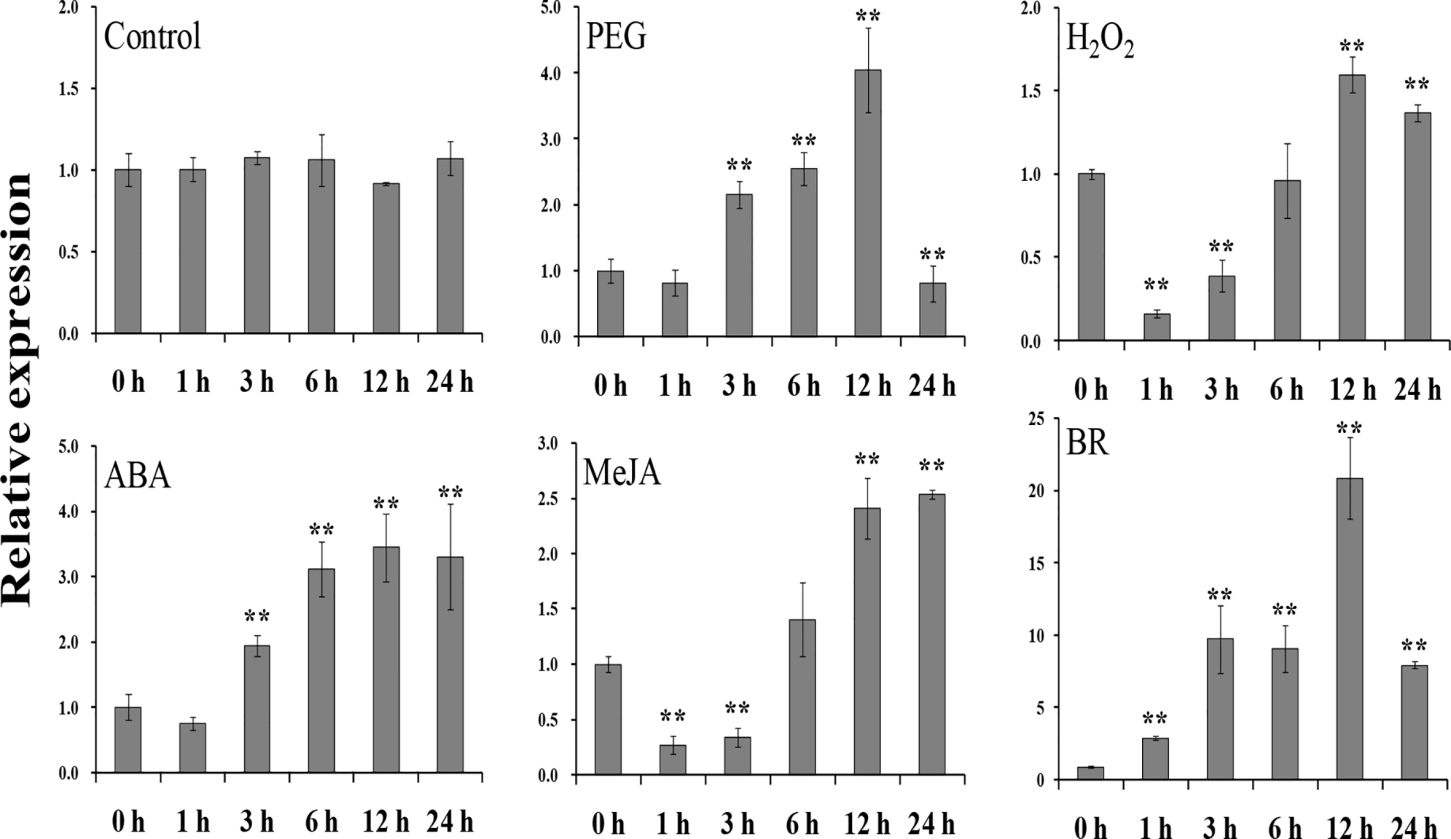
Expression analysis of *IbEGF* in *in vitro*-grown Xushu55-2 plants after different time points (h) in response to H_2_O (control), 30% PEG6000, 100 mM H_2_O_2_,100 mM ABA, 100 μM MeJA and 100 nM BR, respectively. The expression level of *IbEGF* in the plant sampled at 0 h was set to 1. The data are presented as the means ± SEs (n = 3). ** indicates a significant difference from that of the untreated control (0 h) at *P*<0.01 according to Student’s *t*-test.

To study the potential function of *IbEGF* in sweet potato, its expression in different tissues and treatments of Xushu55-2 was analyzed with qRT-PCR. In the *in vitro*-grown Xushu55-2 plants, the root tissue showed the highest expression level of *IbEGF* (**Supplementary Figure S3A**). For the field-grown Xushu55-2 plants, the expression level of *IbEGF* was highest in the stem tissue (**Supplementary Figure S3B**). The expression of *IbEGF* peaked at 12 h (4.90-, 1.30-, 3.44- and 20.86-fold, respectively) after PEG6000, H_2_O_2_, ABA and BR treatments and at 24 h (2.63-fold) after MeJA treatment (**Figure 1**). These results suggest that *IbEGF* might be involved in drought, H_2_O_2_, ABA, MeJA and BR response pathways.

### Subcellular localization

**Figure 2:**
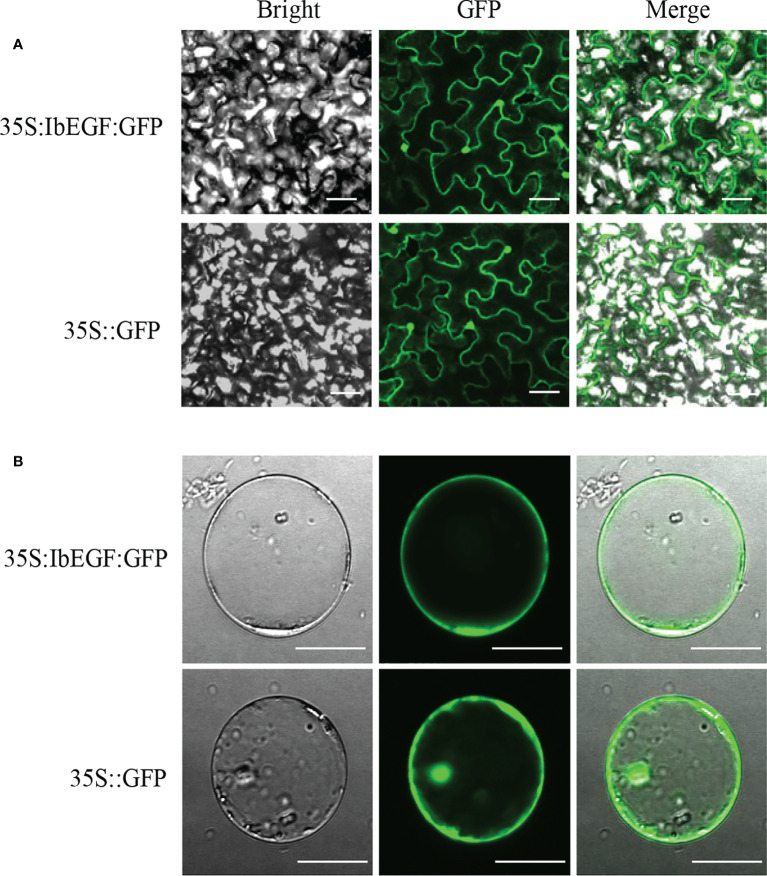
Subcellular localization of IbEGF in tobacco leaf hypodermal cells **(A)** and maize protoplasts **(B)**. Confocal scanning microscopy images showing the localization of IbEGF-GFP to nucleus and cell membrane. The empty pSuper1300 vector (35S:GFP) was used as a control. Bars = 20 μm.

Confocal images taken from the leaf epidermal cells of *N. benthamiana* and the maize protoplasts exhibited that IbEGF-GFP fluorescence was observed in the nucleus and cell membrane (**Figure 2**). These results indicated that IbEGF was localized to the nucleus and cell membrane.

### Transactivation activity

All the yeast cells harbouring pGBKT7-*IbEGF*, pGBKT7-53 (positive control) or pGBKT7 (negative control) grew well on the SD/-Trp medium (**Supplementary Figure S4A**). The yeast cells with pGBKT7-*IbEGF* and negative control did not grow, but the positive control yeast cells showed good growth on medium with X-α-Gal(SD/-Trp/-His/X) (**Supplementary Figure S4B**). These results demonstrated that IbEGF had no transactivation activity.

### Regeneration of the transgenic sweet potato plants

Cell aggregates of Lizixiang (**Supplementary Figure S5A**) cocultivated with EHA 105 carrying pSuper1300-*IbEGF* were cultured on the selective MS medium with 2.0 mg L^-1^ 2,4-dichlorophenoxyacetic acid (2,4-D), 100 mg L^-1^ carbenicillin (Carb) and 10 mg L^-1^ hygromycin (Hyg). After 4 weeks, the 16 Hyg-resistant embryogenic calluses were obtained from the cocultivated 530 cell aggregates (**Supplementary Figure S5B**). These Hyg-resistant embryogenic calluses were transferred to MS medium with 1.0 mg L^-1^ ABA and 100 mg L^-1^ Carb, and after 4 weeks of transfer, they formed plantlets (**Supplementary Figures S5C, D).** Forty-four of 95 regenerated plants were proved to be transgenic by PCR analysis, named L1, L2, …, L44, respectively (**Supplementary Figure S5E**). qRT-PCR analysis revealed that the expression level of *IbEGF* was significantly increased in most of the transgenic plants compared with that of WT (**Supplementary Figure S5F**). These transgenic plants were transplanted to pots and showed a survival rate of 100% in a greenhouse (**Supplementary Figure S5G**).

### Enhanced drought tolerance

**Figure 3:**
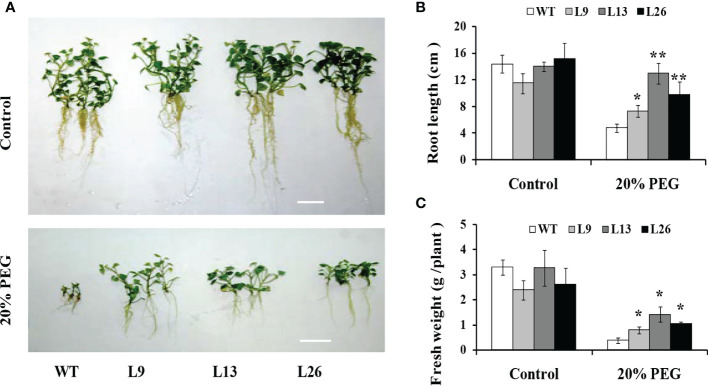
Responses of *in vitro*-grown transgenic sweet potato plants and WT cultured on MS medium without (control) or with 20% PEG6000 for 4 weeks. **(A)** Phenotypes. **(B)** Root length. **(C)** Fresh weight. The data are presented as the mean ± SEs (n = 3). * and ** indicate significant differences from that of WT at *P*<0.05 and *P*<0.01, respectively, according to Student’s *t*-test.

The three transgenic sweet potato plants, L9, L13 and L26, with the higher expression level of *IbEGF*, were selected to test their drought tolerance. Under 20% PEG6000 treatment, the transgenic plants exhibited significantly better growth and rooting, and their fresh weight (FW) was significantly increased compared with WT (**Figures 3A–C**). No difference in growth was observed between the transgenic plants and WT under normal conditions (**Figures 3A–C**).

**Figure 4:**
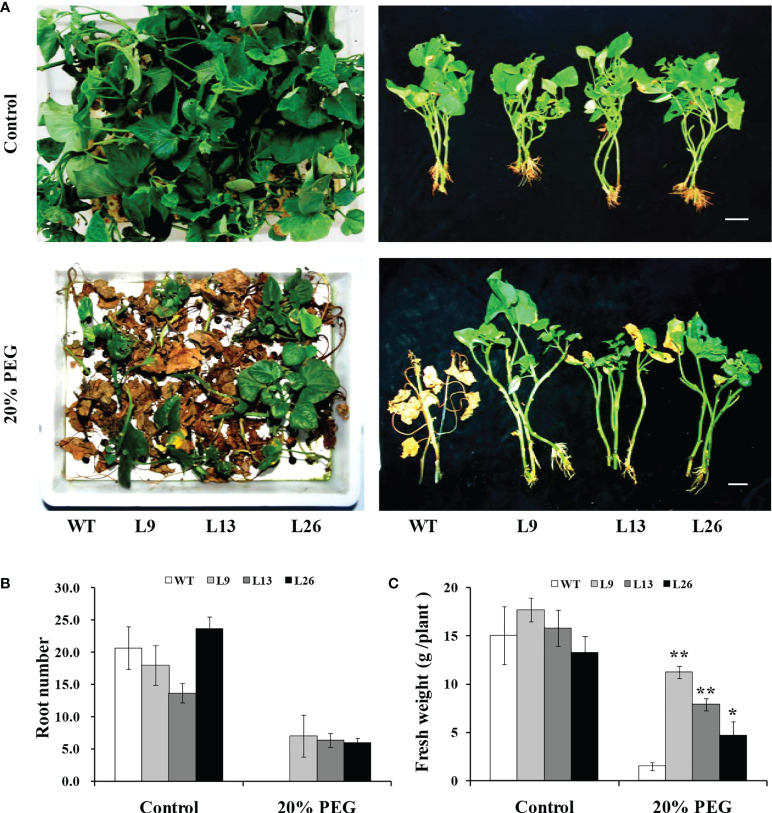
Responses of transgenic sweet potato plants and WT treated in Hoagland solution for 4 weeks (control) or in Hoagland solution with 20% PEG6000 for 2 weeks followed by 2 weeks of Hoagland solution. **(A)** Phenotypes. **(B)** Root number. **(C)** Fresh weight. * and ** indicate significant differences from that of WT at *P*<0.05 and *P*<0.01, respectively, according to Student’s *t*-test.

**Figure 5:**
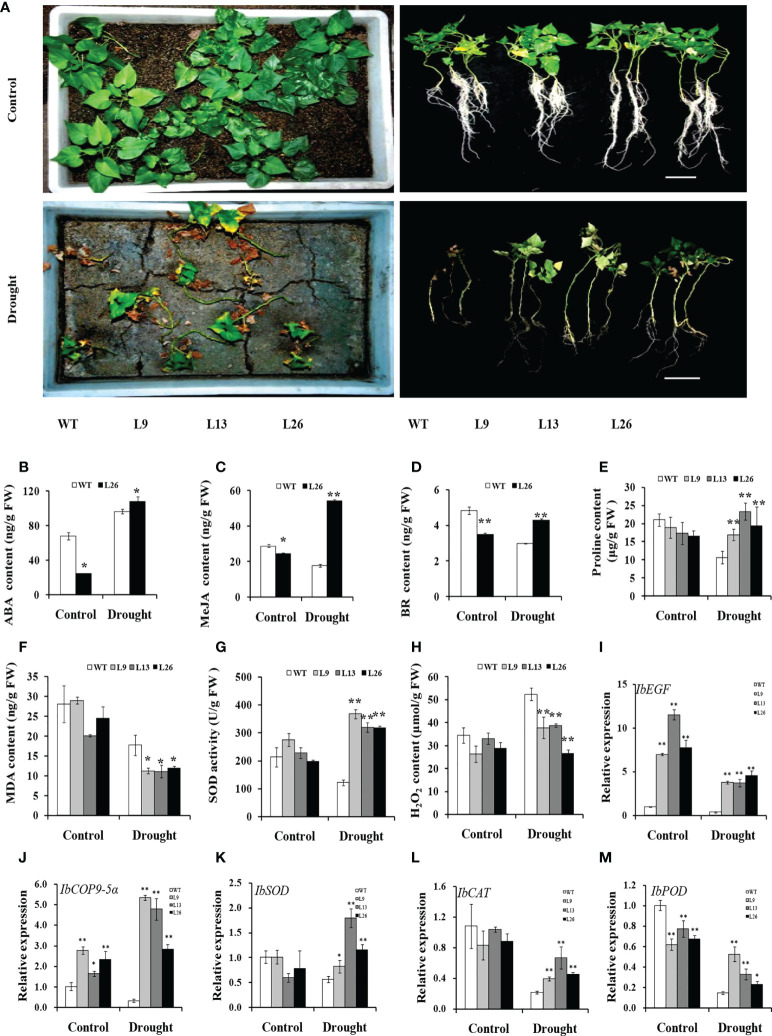
Responses of transgenic sweet potato plants and WT grown in transplanting boxes to drought stress. **(A)** Phenotypes of plants irrigated with Hoagland solution for 8 weeks (control) or stressed by an 8-week-long drought treatment. **(B–H)** ABA content, MeJA content, BR content, proline content, MDA content, SOD activity, and H_2_O_2_ content in the leaves of plants after 4 weeks of treatment, respectively. **(I–M)** Transcript levels of *IbEGF*, *IbCOP9-*5*α*, *IbSOD*, *IbCAT*, and *IbPOD* in the leaves of plants after 4 weeks of treatment, respectively. The transcript levels of the genes in WT under normal treatment were set to 1. * and ** indicate significant differences from that of WT at *P*<0.05 and *P*<0.01, respectively, according to Student’s *t*-test.

To further evaluate drought tolerance, the cuttings from field-grown L9, L13, L26 and WT plants were incubated in Hoagland solution with/without 20% PEG6000. As shown in **Figures 4A–C**, no difference in growth between the transgenic plants and WT was observed in Hoagland solution without PEG6000. However, the growth and rooting of the transgenic plants were significantly better than those of WT in Hoagland solution with PEG6000. Furthermore, the cuttings of the transgenic plants grown in a transplanting box formed new leaves and roots, while WT turned brown to death after drought treatment (**Figure 5A**). Based on these results, the transgenic sweet potato plants exhibited enhanced drought resistance.

### Underlying mechanism of *IbEGF* in drought tolerance

**Figure 6:**
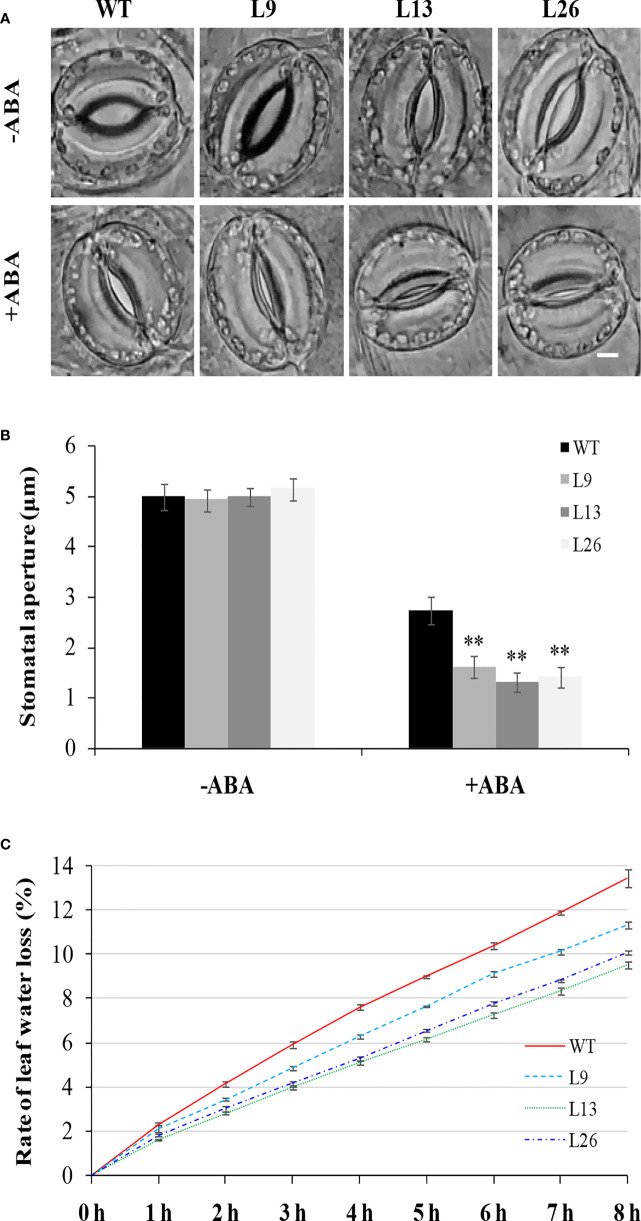
Stomatal aperture and leaf water loss rate of transgenic sweet potato plants and WT. **(A, B)** Stomatal aperture of greenhouse-grown plants under normal condition (-ABA) and treated with 20 μM ABA for 2 h. Bar = 5 μm. Data are presented as the means ± SD (n = 80). ** indicates significant difference from that of WT at *P*<0.01, according to Student’s *t*-test. **(C)** Leaf water loss rate of greenhouse-grown plants.

**Figure 7:**
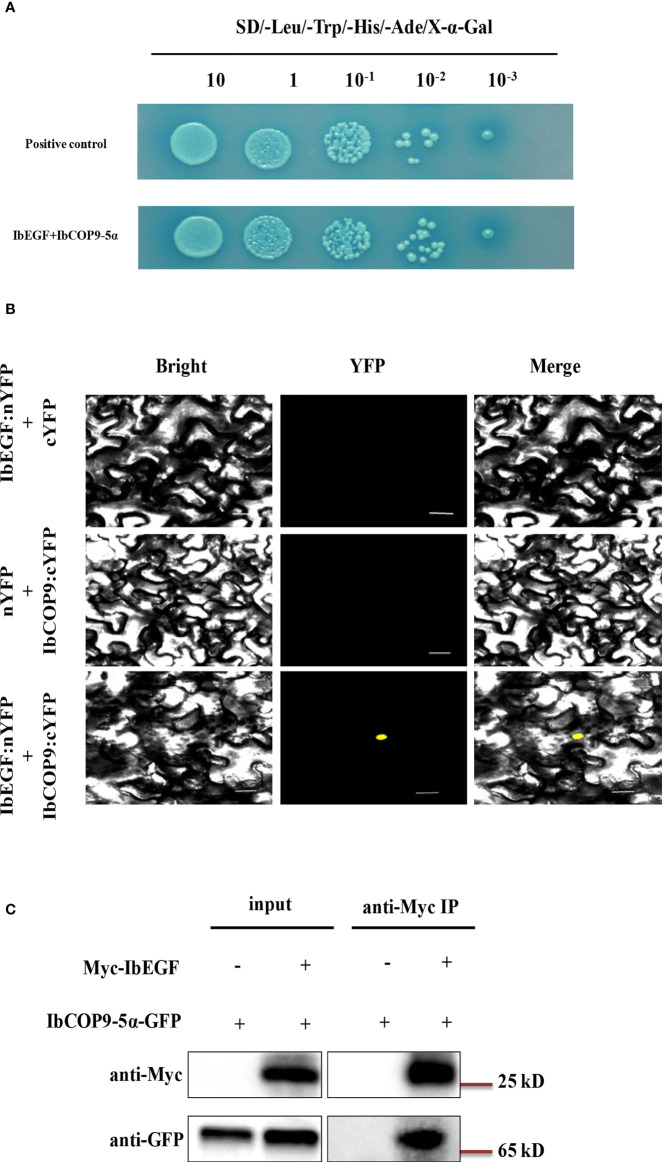
*In vivo* interaction between IbEGF and IbCOP9-5α. **(A)** IbEGF/IbCOP9-5α interaction in Y2H Gold cells by Y2H assay. **(B)** IbEGF/IbCOP9-5α interaction in the tobacco nucleus by BiFC assay. The yellow fluorescent protein (YFP) signals were predominantly localized in the nucleus. Bars = 20 μm. **(C)** IbEGF/IbCOP9-5α interaction by the co-IP assay. Total proteins from *N. benthamiana* leaf cells expressing Myc-IbEGF and IbCOP9-5α-GFP were extracted and incubated with anti-Myc magnetic beads. Proteins before (input) and after IP were detected with anti-Myc and anti-GFP antibodies.

To investigate the underlying mechanism of *IbEGF* in drought tolerance, the components related to drought tolerance were measured. The results showed that the ABA, MeJA, BR and proline contents and SOD activity of the transgenic plants were significantly increased, while their MDA and H_2_O_2_ contents were significantly decreased compared with those of WT under drought stress (**Figures 5B–H**). Further analysis indicated that the expression of *IbCOP9-*5*α*, *IbSOD*, *IbCAT* and *IbPOD* was upregulated in the transgenic plants (**Figures 5I–M**). The transgenic sweet potato plants exhibited reduced ABA-induced stomatal apertures (**Figures 6A, B**) and reduced leaf water loss rate compared with WT (**Figure 6C**). Furthermore, Y2H assays showed that IbCOP9-5α was the potential interacting protein with IbEGF (**Figure 7A**). To further investigate interactions between IbEGF and IbCOP9-5α in plants, BiFC and co-IP assays were performed. The YFP signals were observed in the nucleus of *N. benthamiana* leaf hypodermal cells (**Figures 7B, C**). In a co-IP assay, Myc-IbEGF was co-precipitated by anti-Myc antibody using total proteins extracted from *N. benthamiana* leaves co-expressing IbCOP9-5α-GFP and Myc-IbEGF, but not using total proteins extracted from control leaves expressing IbCOP9-5α-GFP alone (**Figure 7C**). All the results indicated that IbEGF interacted with IbCOP9-5α.

## Discussion

### *IbEGF* enhances drought tolerance

The sORF-encoded polypeptides play crucial roles in plant defense, growth and development, fertilization and environmental stresses including drought, salt, mechanical wounding, pathogen infection and nutrient imbalance (Lindsey et al., 2002; Ryan et al., 2002; Matsuzaki et al., 2010; Nakaminami et al., 2018; Takahashi et al., 2018; Chen et al., 2020). OsDT11 and OsDSSR1 in rice and CLE25 and CLE9 in *Arabidopsis* positively regulate responses to drought stress (Li et al., 2017; Cui et al., 2018; Takahashi et al., 2018; Zhang et al., 2019). However, the functions of the EGF protein family with EGF_CA domain in plants are still unclear.

In the present study, the *IbEGF* gene was isolated from sweet potato. Its ORF encoded a polypeptide of 83 aa (**Supplementary Figure S1**). This is the first reported sORF with the EGF_CA domain in sweet potato. The expression of *IbEGF* was significantly upregulated under PEG stress (**Figure 1**). Its overexpression significantly enhanced drought tolerance of the transgenic sweet potato plants (**Figures 3** **–** **5**). It is thought that *IbEGF* is a novel sORF gene involved in drought tolerance of sweet potato.

### *IbEGF* positively regulates ABA, JA and BR signalling pathways

Drought causes oxidative stress and metabolic and osmotic damage and inhibits plant growth (Fàbregas and Fernie, 2019). Plants evolve complex regulatory hormonal signalling networks for adapting to drought conditions (Miller et al., 2010). Drought triggers the accumulation of ABA, JA and BR in plant tissues, which leads to drought tolerance (Rohwer and Erwin, 2008; Daszkowska-Golec, 2016; Zhou et al., 2020). ABA emerges as a crucial regulator of the drought response (Li et al., 2021). JA plays an important role as a signal molecule that induces tolerance mechanisms under drought stress (Rohwer and Erwin, 2008). BR regulates adaptations to cope with drought stress (Planas-Riverola et al., 2019).

As a new class of plant hormones, the sORF-encoded polypeptides play an essential role in responses to changes in the environments by modulating other plant hormones (Ryan and Pearce, 2001; Chen et al., 2020; Maurel et al., 2021). It was reported that *OsDT11* in rice and *CLE25* and *CLE9* in *Arabidopsis* reduced stomatal density or aperture and enhanced drought tolerance by ABA signalling (Li et al., 2017; Takahashi et al., 2018; Zhang et al., 2019). There was crosstalk between peptide hormone BoPep4 and ABA signalling pathways under salinity stress (Wang et al., 2022a). The serine rich endogenous peptide (SCOOP) increased defense against a generalist herbivore by modulating the JA pathway in *Arabidopsis* (Stahl et al., 2022).

COP9 signalosome is a highly conserved transcriptional regulator (Chamovitz, 2009; Jin et al., 2014). Several findings have showed that COP9 signalosome regulates plant response to abiotic stresses such as oxidative stress, salt stress and high-temperature stress through the ABA, JA, gibberellic acid (GA), auxin and ROS signalling pathways (Nahlik et al., 2010; Singh and Chamovitz, 2019; Wang et al., 2022b). COP9-5α (CNS5A) is an essential player in the regulation of plant development and stress (Singh et al., 2021). In *Arabidopsis*, CSN5A regulated seed germination and salt tolerance by ABA signalling (Jin et al., 2018; Zhou et al., 2021). IbGATA24 interacting with IbCOP9-5α positively regulated drought and salt resistance by ABA and ROS signalling in transgenic *Arabidopsis* (Zhu et al., 2022).

**Figure 8:**
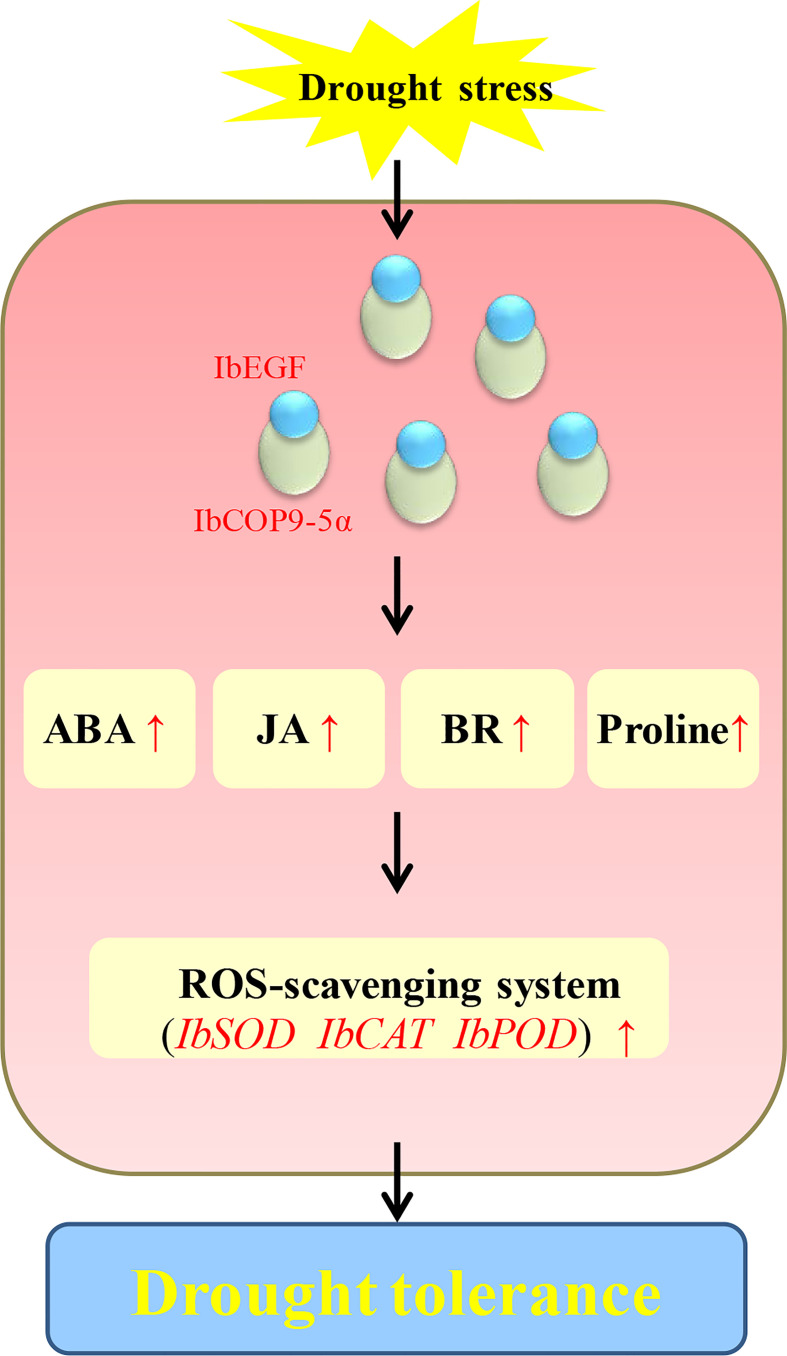
Proposed working model of *IbEGF* in the transgenic sweet potato plants to drought stress.

Our results showed that the *IbEGF* gene was upregulated after treatments of ABA, MeJA and BR, respectively (**Figure 1**). The *IbEGF*-overexpressing sweet potato plants exhibited better growth and more accumulation of ABA, MeJA and BR under drought stress (**Figures 3** **–** **5**). Further analysis indicated that the expression level of *IbCOP9*-*5α* was increased in the transgenic sweet potato plants under drought stress (**Figure 5**). Y2H, BiFC and co-IP assays demonstrated that IbEGF interacted with COP9-*5α* (**Figures 7**). Our previous study indicated that IbCOP9-5α is a positive regulator of the response to drought stress and the *IbCOP9-5α*-overexpressing *Arabidopsis* plants exhibited significantly enhanced drought tolerance (Zhu et al., 2022). Therefore, it is thought that IbEGF with the help of COP9-5α could positively regulate the ABA, JA and BR signalling pathways under drought stress, which results in enhanced drought tolerance (**Figure 8**).

### *IbEGF* Positively Modulates Proline Accumulation and ROS-Scavenging System

The overproduction of ROS in plants causes damage to proteins, lipids, carbohydrates and DNA under drought, salinity and heat stresses (Gill and Tuteja, 2010; Choudhury et al., 2017; Waszczak et al., 2018). More proline accumulation can protect plants from drought stress and ROS damage (Phang et al., 2010; Liu et al., 2014; Adamipour et al., 2020). In rice, *OsDSSR1* was induced by drought, salinity, ABA and H_2_O_2_ treatments and its overexpression enhanced drought tolerance by increasing the accumulation of free proline and soluble sugars and further promoting *OsSodCc2* and *OscAPX* expression and SOD and ascorbate peroxidase (APX) activities (Cui et al., 2018).

In our study, overexpression of *IbEGF* increased the proline content and SOD activity and reduced the H_2_O_2_ level in transgenic sweet potato under drought stress (**Figure 5**). *IbSOD*, *IbCAT* and *IbPOD* were also upregulated (**Figure 5**). It is suggested that overexpression of *IbEGF* enhances drought tolerance by positively modulating proline accumulation and ROS-scavenging system in transgenic sweet potato (**Figure 8**). Based on all the above results, we propose that IbEGF interacting with IbCOP9-5α positively regulates the hormone signalling pathways and proline biosynthesis and further activates the ROS scavenging system, which lead to enhanced drought tolerance in transgenic sweet potato (**Figure 8**).

## Conclusion

A novel sORF gene, *IbEGF*, was isolated and characterized from sweet potato. Its overexpression in sweet potato enhanced tolerance to drought stress, increased contents of ABA, MeJA, BR and proline and activity of SOD, decreased levels of MDA and H_2_O_2_ and upregulated expression of *IbCOP9-5α*, *IbSOD*, *IbCAT* and *IbPOD*. It is suggested that IbEGF interacting with IbCOP9-5α enhances drought tolerance by positively regulating the hormone signalling pathways, increasing proline accumulation and further activating the ROS scavenging system in transgenic sweet potato.

## Data availability statement

The original contributions presented in the study are included in the article/**Supplementary Material**. Further inquiries can be directed to the corresponding author.

## Author contributions

QL and YZ conceived and designed the experiments. YZ performed the experiments. YZ and HoZ analyzed the data. QL, SX, ZW, SH, HuZ, SG and NZ contributed reagents, materials and analysis tools. QL and YZ wrote the paper. All authors contributed to the article and approved the submitted version.

## Funding

This study was supported by the National Key R&D Program of China (grant number 2018YFD1000704/2018YFD1000700) and the earmarked fund for CARS-10-Sweetpotato.

## Conflict of interest

The authors declare that the research was conducted in the absence of any commercial or financial relationships that could be construed as a potential conflict of interest.

## Publisher’s note

All claims expressed in this article are solely those of the authors and do not necessarily represent those of their affiliated organizations, or those of the publisher, the editors and the reviewers. Any product that may be evaluated in this article, or claim that may be made by its manufacturer, is not guaranteed or endorsed by the publisher.
