# Supplementary material for: A novel small open reading frame gene, IbEGF, enhances drought tolerance in transgenic sweet potato
Source: Front Plant Sci. 2022 Oct 31;13:965069. doi: 10.3389/fpls.2022.965069 (PMC9660231; doi:10.3389/fpls.2022.965069)
Supplement: Supplementary file 1 [file DataSheet_1.doc]

**FIGURE S1** | Sequence alignment **(A)** and phylogenetic tree **(B)** of IbEGF with its homologs from other plants.

**FIGURE S2** | Promoter of *IbEGF* showing different *cis*-acting regulatory elements associated with abiotic stress responses.

**FIGURE S3** | The transcript levels of *IbEGF* in different tissues of the *in vitro*-grown **(A)** and field-grown **(B)** plants of Xushu55-2. L: Leaf; S: Stem; R: root; HR: Hairy root; PR: Pencil root; SR: Storage root. The transcript level of *IbEGF* in the leaf tissue was set to 1. The data are presented as the means ± SEs (n = 3). The different small letters indicate a significant difference at *P* <0.05 according to Student’s *t*-test.

**FIGURE S4** | Transactivation activity assay of IbEGF in yeast. (**A**) Transformed yeast cells harbouring different expression vectors cultured on SD/-Trp medium. (**B**) Transformed yeast cells harbouring different expression vectors cultured on SD/-Trp/-His medium with X-α-Gal. pGBKT7 (-) and pGBKT7-53 (+) were used as negative and positive controls, respectively.

**FIGURE S5** | Production of the *IbEGF*-overexpressing sweetpotato plants. **(A)** Embryogenic suspension cultures proliferating in MS medium with 2.0 mg L^-1^ 2,4-D. **(B)** Hyg-resistant calluses formed after 4 weeks of selection on MS medium with 2.0 mg L^-1^ 2,4-D, 100 mg L^-1^ Carb and 10 mg L^-1^ Hyg. **(C)** Germination of somatic embryos from Hyg-resistant calluses on MS medium with 1.0 mg L^-1^ ABA and 100 mg L^-1^ Carb. **(D)** Whole regenerated plantlets. **(E)** PCR analysis of the transgenic plants. Lane M: BL2000 plus DNA marker; Lane W: Water; Lane P: plasmid pSupper1300-*IbEGF* as a positive control; Lane WT: Lizixiang plant as a negative control. **(F)** Expression analysis of *IbEGF* in the transgenic plants.** indicates a significant difference from that of WT at *P*<0.01 according to Student’s *t*-test. **(G)** The transgenic plants grown in a greenhouse.

**Table S1** | Primers used in this study.

| Primer name | Primer sequence (5’-3’) |
| --- | --- |
| Primers for 5’-promoter region |  |
| IbEGF-Pro-F | ATTATTGAACTATGCCCATCAG |
| IbEGF-Pro-R | GTTTGGTTGTGTGTTTGCAAG |
| Primers for vector construction | |
| IbEGF-F | ATGGCCTCCCATAATGCTT |
| IbEGF-R | TCATGTCGATTCAACCGACT |
| *IbEGF-*OS-F(*Xba*Ⅰ) | GCTCTAGAATGGCCTCCCATAATGCTT |
| *IbEGF-*OS-R(*Pst*Ⅰ) | AACTGCAGTGTCGATTCAACCGACT |
| *IbEGF*-BD-F-*(Eco* RⅠ*)* | GGAATTCATGGCCTCCCATAATGCTT |
| *IbEGF*-BD-R-(*Sal* I) | GCGTCGACTCATGTCGATTCAACCGACT |
| *IbCOP9-AD*-F(*Eco* RⅠ) | GGAATTCATGTATTTACAGGCGGCACAGT |
| *IbCOP9*-AD-R(*Bam* HⅠ) | CGGGATCCCGTTTCAACCATGGGC |
| *IbCOP9-*CE-F(*Asc* I) | GGCGCGCCATGTATTTACAGGCGGCACAGT |
| *IbCOP9*-CE-R(*Kpn* I) | GGGGTACCTGTCGATTCAACCGACT |
| *IbEGF*-NE-F(*Asc* I) | GGCGCGCCATGGCCTCCCATAATGCTT |
| *IbEGF*-NE-R(*Kpn* I) | GGGGTACCTGTCGATTCAACCGACT |
| Primers for transformant identification | |
| pSuper-1300-F | GACGCCATTTCGCCTTTTCA |
| pSuper-1300-R | TGAACTTGTGGCCGTTTACGTC |
| Primers for qRT-PCR | |
| *Ibactin*-F | AGCAGCATGAAGATTAAGGTTGTAGCAC |
| *Ibactin*-R | TGGAAAATTAGAAGCACTTCCTGTGAAC |
| *IbEGF*-F | AACCGCCACGATCACATCTC |
| *IbEGF*-R | GAAAGATCCCTGGTGGGTCG |
| *IbCOP9*-*5α***-**F | CATATTCTACTACGACGAGACGG |
| *IbCOP9*-*5α*-R | GGGCGTGGACAACCATCT |
| *IbSOD*-F | TCCTGGACCTCATGGATTTC |
| *IbSOD*-R | GCCACTATGTTTCCCAGGTC |
| *IbCAT*-F | ACGCAATTCCCGGACGTGAT |
| *IbtCAT*-R | AAGCCTTCCATGTGGCGGTA |
| *IbPOD*-F | TTCACGACTGCTTCGTTGA |
| *IbPOD*-R | TTCTCAACCGCGGTCTTAA |
